# Factors that determine catastrophic expenditure for tuberculosis care: a patient survey in China

**DOI:** 10.1186/s40249-016-0100-6

**Published:** 2016-01-25

**Authors:** Chengchao Zhou, Qian Long, Jiaying Chen, Li Xiang, Qiang Li, Shenglan Tang, Fei Huang, Qiang Sun, Henry Lucas

**Affiliations:** Department of Health Service Management and Maternal and Child Health, School of Public Health, Shandong University, Jinan, China; Duke Global Health Institute, Duke University, Durham, NC USA; Global Health Research Center, Duke Kunshan University, Kunshan, China; School of Health Policy and Management, Nanjing Medical University, Nanjing, China; School of Medicine and Health Management, Huazhong University of Science and Technology, Wuhan, China; School of Public Health, Xi’an Jiaotong University, Xi’an, China; National Center for TB Control and Prevention, China CDC, Beijing, China; Center for Health Management and Policy, Shandong University, Wen-hua-xi Road No.44, Jinan City, 250012 China; Key Lab of Health Economic and Policy Studies of Ministry of Health, Jinan, China; Institute of Development Studies, Sussex University, Brighton, UK

**Keywords:** Catastrophic health expenditure, Tuberculosis, Determinants, China

## Abstract

**Background:**

Tuberculosis (TB) often causes catastrophic economic effects on both the individual suffering the disease and their households. A number of studies have analyzed patient and household expenditure on TB care, but there does not appear to be any that have assessed the incidence, intensity and determinants of catastrophic health expenditure (CHE) relating to TB care in China. That will be the objective of this paper.

**Methods:**

The data used for this study were derived from the baseline survey of the China Government – Gates Foundation TB Phase II program. Our analysis included 747 TB cases. Catastrophic health expenditure for TB care was estimated using two approaches, with households defined as experiencing CHE if their annual expenditure on TB care: (a) exceeded 10 % of total household income; and (b) exceeded 40 % of their non-food expenditure (capacity to pay). Chi-square tests were used to identify associated factors and logistic regression analysis to identify the determinants of CHE.

**Results:**

The incidence of CHE was 66.8 % using the household income measure and 54.7 % using non-food expenditure (capacity to pay). An inverse association was observed between CHE rates and household income level. Significant determinants of CHE were: age, household size, employment status, health insurance status, patient income as a percentage of total household income, hospitalization and status as a minimum living security household. Factors including gender, marital status and type of TB case had no significant associations with CHE.

**Conclusions:**

Catastrophic health expenditure incidence from TB care is high in China. An integrated policy expanding the free treatment package and ensuring universal coverage, especially the height of UHC for TB patients, is needed. Financial and social protection interventions are essential for identified at-risk groups.

**Electronic supplementary material:**

The online version of this article (doi:10.1186/s40249-016-0100-6) contains supplementary material, which is available to authorized users.

## Multilingual abstracts

Please see Additional file 1 for translations of the abstract into the six official working languages of the United Nations.

## Background

Protecting people from financial risk associated with ill health is a desirable objective of health policy worldwide [1–4]. Such risk can be quantified in terms of catastrophic health expenditures (CHE). Catastrophic health expenditures is defined as out-of-pocket expenditure for health care that exceeds a specified proportion of household income, with the consequence that the household may have to sacrifice the consumption of other goods and services necessary for their well-being [1, 5]. Catastrophic health expenditures does not necessarily mean high health care costs. Relatively small expenditures for common illnesses may have serious financial implications for poor households [1, 6–9]. Over recent years, the World Health Organization (WHO) has promoted the concept of universal health coverage (UHC), emphasizing the need for access to services at an affordable cost to protect households from CHE [10].

Tuberculosis (TB) has significant economic impacts in many countries and may hamper national development [1, 11–16]. China has the second largest national burden of TB cases. In 2012, an estimated 1.0 million TB cases were diagnosed (range 0.9–1.1 million) and there were 44,000 deaths from the disease (range, 43,000–45,000) [17]. Tuberculosis is most prevalent among the 15–54 age group, which is the most economically productive sector of the population [18, 19]. The disease can therefore cause enormous economic and social disruption by reducing both labor supply and productivity.

The economic effects of TB affect not only national economies, but also individuals and households [20]. In China, the government provides free smear tests for TB suspects and a basic treatment package for TB cases. In theory, TB can be diagnosed and treated without any out-of-pocket health care expenditures by patients or their households. However, many studies have shown that there are often many associated health care costs, including payment for ancillary drugs, for example for liver-protection, and extra diagnostic tests, as well as considerable non-medical costs, including expenditures for transport and accommodation [21–23]. Furthermore, patients and other household members who care for them may suffer reduced incomes due to lower productivity and/or loss of employment opportunities, and may experience the intangible costs related to the social stigma associated with their illness and the potential breakdown of the family unit [24].

A number of studies have analyzed patient and household expenditure on TB care in China [16, 21–23] but there does not appear to be any that have assessed the associated incidence, intensity and determinants of CHE. The present study’s overall goal is to describe the profile of CHE among TB patients in China. To do so, we have several specific objectives. First, we will estimate the extent of CHE for TB care in China. Second, we will identify the associated household socio-demographic and economic factors, with the aim of recommending policies that can reduce the economic burden of TB on patients and their households.

## Methods

### Data source

Since 2009, the Gates Foundation, in collaboration with the Chinese Ministry of Health/China CDC, has been implementing an innovative program on TB/multidrug-resistant TB (MDR-TB) control and prevention in four Chinese cities [25]. In 2013, the second phase of this program was initiated. The program aims to use innovative tools and delivery approaches to establish comprehensive TB/MDR-TB control models which can be scaled up over time by the National TB Prevention and Control Program. The data used for this study were derived from the baseline studies conducted for this second phase.

### Study sites

**Fig. 1:**
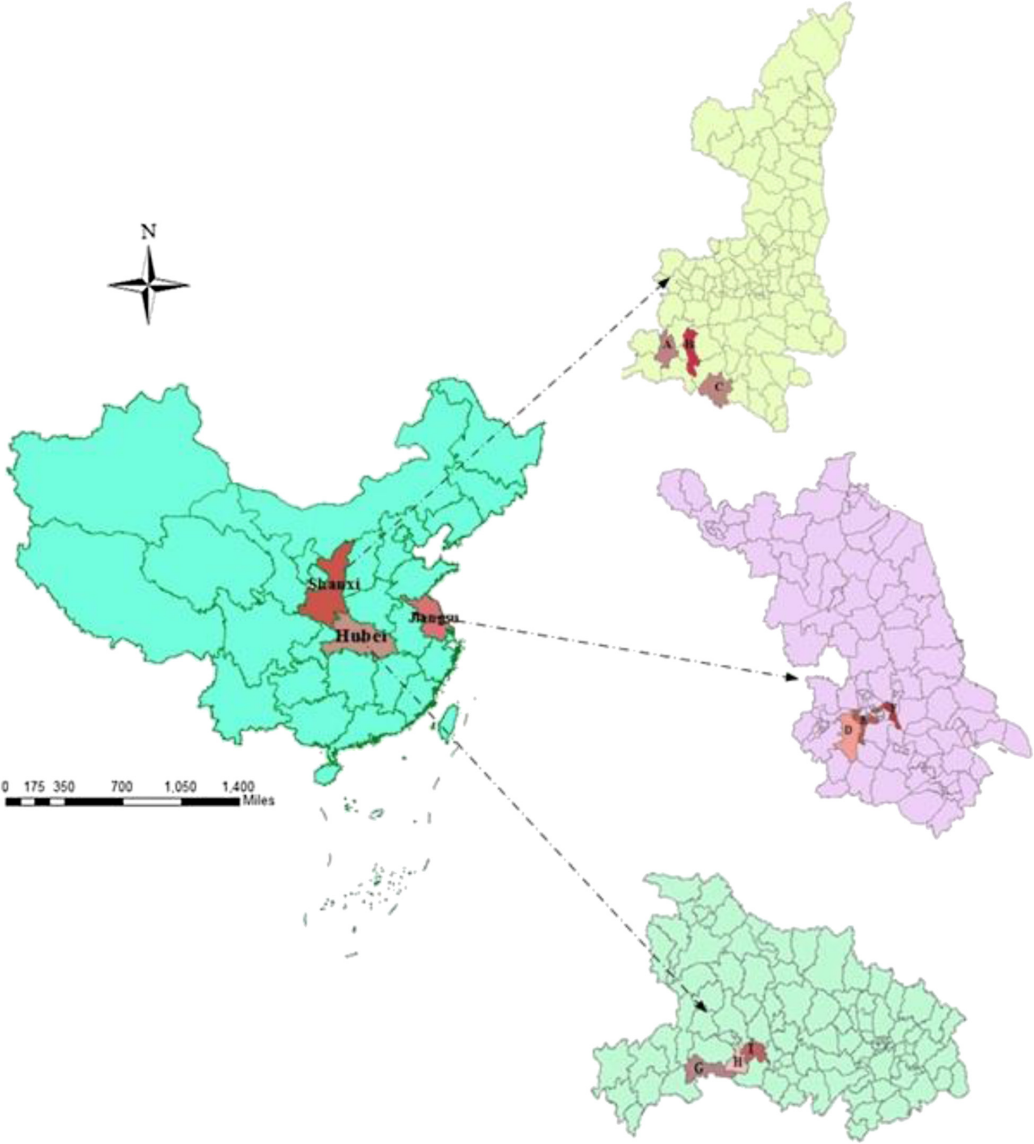
Location of nine study sites in China

The baseline studies were conducted in three cities (Zhenjiang City, Jiangsu Province; Yichang City, Hubei Province; and Hanzhong City, Shaanxi Province), which are geographically located in the eastern, central and western regions of China. Three counties or districts (one from each category of high, middle and low GDP per capita) were then selected as study sites in each city (Dantu, Yangzhong and Jurong in Zhenjiang; Zhijiang, Yidu and Wufeng in Yichang; and Chenggu, Mian and Zhenba in Hanzhong). Figure 1 shows the location of these sites. TB dispensaries and designated hospitals were the institutions authorized to provide TB diagnosis, treatment and case management. All newly diagnosed TB cases were required to be registered in the local dispensary or designated hospital and reported to upper level health authorities.

### Study participants

A cluster sampling method based on townships/streets was used to recruit TB patients. Using selected key indicators (financial burden of TB care and treatment adherence), the minimum required sample size per city was calculated to be 264 cases. Three townships/streets were selected in each county/district using probability proportional to size (PPS) sampling and 30 TB cases were randomly sampled in each, using the list of registered cases and adopting the criteria that each patient had completed or stopped treatment during 2012. The sample included both new patients and those who had been previously treated for TB but where treatment had failed or the patient had defaulted or relapsed.

A total of 797 TB patients were interviewed, of whom 50 failed to report out-of-pocket expenditures or household income. Thus, 747 cases with complete data were included in the analysis.

### Data collection

The baseline survey was conducted between April and May 2013. All of the participants were interviewed after treatment completion/interruption. The TB patients were interviewed face-to-face at local TB dispensaries or designated hospitals using a standardized survey questionnaire which included personal demographic and socio-economic information (age, sex, education, household income/expenditure, etc.), direct health service expenditures, and non-medical expenses (transportation and accommodation costs, etc.). The household income and expenditure (e.g. food expenditure) were ascertained through direct questions.

The patient survey was conducted by university/college students from Huazhong University of Science and Technology (Yichang), Xi’an Jiaotong University (Hanzhong) and Nanjing Medical University (Zhejiang). A technical assistance team (TA) from the Duke Global Health Institute, USA, the Institute of Development Studies, UK and Shandong University, China also took part in the baseline survey. The interviewers received training on interview skills and the contents of the questionnaire before the survey.

### Measuring CHE and definitions

CHE is usually assessed by incidence (headcount) and intensity indicators, which were described in detail in Wagstaff et al. [26, 27]. Measuring CHE requires specification of the thresholds for household income or capacity to pay (non-food expenditure), which household health expenditure should not exceed. There is no consensus on these thresholds, but the two most commonly used are: 10 % of total household income; and 40 % of household capacity to pay [28]. In this study, CHE for TB care was estimated using both criteria.

Out-of-pocket (OOP) payments for TB care include direct health expenditures on diagnosis and treatment (consultation fees, laboratory tests, X-rays, drugs, and hospital care) and associated non-medical expenses (transport and accommodation costs for patient and companions, nutrition supplement costs), but not income loss. The headcount indicator (H) is the percentage of households whose OOP payments (numerator) as a proportion of household income or non-food expenditure (denominator) exceeds a given threshold. The mean gap indicator (G) is the average amount by which payments, as a proportion of household income (or non-food expenditure), exceed this threshold [26, 27]. Finally, the mean positive gap (MPG) is equal to G/H, the excess expenditure per household experiencing CHE.

### Data management and analysis

In order to ensure quality, the double entry data verification approach was adopted using EPI Data 6.04. The statistical package SPSS 13.0 was used to analyze the data. Household income, household expenditure, OOP and capacity to pay were presented as means (Standard deviation, SD) and medians (percentile 25 and percentile 75, p25 and p75) to allow for the expected positively skewed distributions. Sensitivity analysis of the incidence and intensity of CHE for TB care was applied using different thresholds across different household income groups. Chi-square tests were used to identify factors associated with CHE. Multivariate logistic regression analysis was employed to assess the effects of explanatory variables in a multivariate model. Two multivariate models were run respectively using both of the above-mentioned definitions. Statistical significance was set at 5 %.

### Quality assurance

The questionnaire and survey procedures were tested in a pilot study carried out in Zhenjiang city in March 2013 and then refined before use in the three project cities. Key researchers from the above four Chinese universities acted as survey supervisors to check the consistency and quality of the data collected.

### Ethical consideration

The Ethical Committee of China CDC reviewed and approved the study protocols and instruments. Informed consents were obtained from all study participants.

## Results

### Household income/expenditure, capacity to pay and OOP payments for TB

The mean annual household income was US$4962^1^ and expenditure US$3641 (median values US$3414 and US$3185). The mean capacity to pay was US$2384 and the median US$1592. The mean OOP payment for TB was US$1156 (median value US$637), 23 % of the average annual household income and 49 % of the mean capacity to pay. About 45 % of total OOP was spent on hospitalization, and over 20 % of this amount was spent before TB diagnosis (see Tables 1 and 2).

**Table 1:** Distribution of direct costs and incidence of catastrophic expenditure for TB, China 2012

Indicators	Study locations			All
	Hanzhong	Yichang	Zhenjiang	
Frequency	236	261	250	747
Average OOP^a^ costs of TB care (US$)^b^				
Mean(SD.)	1139(2376)	847(1822)	1495(1801)	1156(1678)
Median(p25,p75)	478(398,875)	462(292,593)	876(477,2547)	637(318,1273)
Average annual household income (US$)				
Mean(SD.)	3540(4037)	4070(4287)	7236(6612)	4962(5358)
Median(p25,p75)	2389(796,4777)	3185(1455,5175)	6162(2002,10143)	3414(1269,6407)
Average annual household expenditure (US$)				
Mean(SD.)	3045(3140)	3613(3169)	4221(3888)	3641(3449)
Median(p25,p75)	2384(1194,3662)	3185(1393,4777)	3185(1592,5573)	3185(1560,4777)
Average capacity to pay (US$)^c^				
Mean(SD.)	2042(2839)	2494(2761)	2584(3210)	2384(2945)
Median(p25,p75)	1306(557,2433)	1911(804,3463)	1592(796,3184)	1592(716,3001)
OOP costs share of household income (%)	32.2	20.8	20.7	23.3
OOP costs share of capacity to pay (%)	55.8	34.0	57.9	48.5
Households with catastrophic expenditure (%)				
I = >10 % of household income	67.8	65.1	67.6	66.8
II = ≥40 % of capacity to pay	55.8	45.1	63.7	54.7

Note: US$ = United States dollar

a: OOP: out-of-pocket

b: A currency exchange rate of Chinese RMB 628 Yuan to US$1 00 Yuan (at the end of 2012)

c: Capacity to pay means that household expenditure minus food expenditure

**Table 2:** Household direct costs for TB care in different period or services, China 2012

Costs in different period(services)	Hanzhong		Yichang		Zhenjiang		ALL	
	Mean^a^	HHI(%)^b^	Mean	HHI(%)	Mean	HHI(%)	Mean	HHI(%)
Pre-Diagnosis	349	9.9	142	3.5	224	3.1	235	4.7
Post-Diagnosis, Pre-Treatment	15	0.4	19	0.5	55	0.8	30	0.6
In-Patient	513	14.5	409	10.1	649	9.0	522	10.5
Out-Patient	97	2.7	252	6.2	194	2.7	183	3.7
Nutrition Supplementation	166	4.7	25	0.6	373	5.2	186	3.7
Total	1140	32.2	847	20.8	1495	20.7	1156	23.3

a: US$, a currency exchange rate of Chinese RMB 628 Yuan to US$100 Yuan( at the end of 2012)

b: Percentage of mean annual household income

### Catastrophic health expenditure for TB care

Table 3 presents the incidence and intensity indicators relating to CHE for TB care. It shows an inverse association between CHE rates and households income levels. Over 94 % of households in the poorest quintile (Q1) spent at least 10 % of their income directly on TB care as compared to 43 % of those in the richest quintile (Q4). Similar trends were observed when CHE was defined with respect to capacity to pay. Table 3 also demonstrates how CHE rates vary across different thresholds. Almost 67 % of households spent at least 10 % of their household income on TB treatment, 42 % spent at least 25 %, and 31 % spent at least 40 %. Similarly, almost 55 % of households incurred CHE for TB care using a threshold of 40 % of non-food expenditure and this increased to 87 % if the threshold was set to 10 %.

**Table 3:** Incidence and intensity of catastrophic health expenditure for TB care by household economic status, China 2012

Catastrophic expenditure	% of household income			% of non-food expenditure		
	10 %	25 %	40 %	10 %	25 %	40 %
Head Count (HC,%)						
Q1^a^	94.1	83.2	71.8	94.8	82.2	71.2
Q2	82.6	48.8	28.9	90.8	75.6	63.9
Q3	63.4	30.1	19.6	84.1	59.6	50.3
Q4	41.3	15.9	8.9	81.5	55.1	40.8
Total	66.8	42.3	31.3	87.1	66.5	54.7
*P*-value	**0.000**	**0.000**	**0.000**	**0.000**	**0.000**	**0.000**
Mean Catastrophic Payment Gap (%)						
Q1	111.2	98.0	86.9	117.4	104.3	93.2
Q2	32.4	22.0	16.4	67.6	55.5	45.4
Q3	22.6	15.2	11.5	55.9	45.6	37.5
Q4	9.1	5.1	3.3	51.9	41.8	34.7
Total	40.8	32.8	27.7	72.0	60.9	52.1
Mean Positive Gap (%)						
Q1	119.3	121.7	129.6	124.5	129.6	136.3
Q2	39.3	45.6	58.0	74.9	74.6	73.3
Q3	35.6	50.5	58.8	66.6	77.3	75.4
Q4	22.0	32.4	37.7	63.7	76.2	85.7
Total	62.2	81.8	96.3	83.2	93.2	98.3

Note: The bold values indicate statistical significance at 5 % level. a: Quartile 1 (Q1) is the poorest and Quartile 4 (Q4) is the richest

The intensity of CHE for TB care is presented in Table 3 using the mean gap and mean positive gap indicators. On average, health care payments for TB were 41 % higher than the 10 % threshold. For households that experienced CHE, the mean positive gap measure indicated that this excess increases to 62 %. Table 3 also provides these intensity indicators based on a range of income and non-food expenditure thresholds.

### Catastrophic health expenditure distribution

In Table 4, CHE rates were compared across a range of patient or household groups. For households, we found that a higher risk of experiencing CHE was incurred by those in rural areas, those with less than four members and those who received the government ‘minimum living security’ (a group known as “dibaohu” in Chinese, which is identified as a low-income household that is subsidized by the local bureau of civil affairs). Households with patients who were older, who had lower educational levels, who were unemployed, whose incomes accounted for over 50 % of household income, or who were hospitalized during treatment, also tended to incur CHE. There was also a positive relationship between CHE and New Cooperative Medical Scheme (NCMS) membership, but it should be noted that the numbers in other schemes were relatively small and that the majority of NCMS members are in rural households.

**Table 4:** Relationship between patients characteristics and rate of catastrophic expenditures for TB, China 2012

Variable	Rate of I^a^ (n = 747)			Rate of II (n = 728)^b^		
	n (%)	Χ^2^	*P*	n (%)	Χ^2^	*P*
Age(years)		23.07	**0.000**		27.66	**0.000**
≤40	56(48.7)			43(38.7)		
41–59	191(66.6)			139(49.3)		
≥60	252(73.0)			216(64.5)		
Gender		1.14	0.286		0.28	0.600
Male	382(67.9)			296(54.1)		
Female	117(63.6)			102(56.4)		
Type of TB case		3.39	0.066		0.60	0.437
New or never treated	390(65.2)			314(54.0)		
Relapse or previously treated	109(73.2)			84(57.5)		
Residence		5.06	**0.025**		0.36	0.549
Rural	468(67.9)			369(55.0)		
Urban	31(53.4)			29(50.9)		
Education		12.90	**0.005**		14.31	**0.003**
None	108(75.0)			84(61.3)		
Primary school	175(70.9)			149(61.3)		
Junior school	161(61.9)			122(48.0)		
Senior school or above	55(57.3)			43(45.7)		
Marital status		3.58	0.167		4.46	0.108
Single	399(66.3)			319(54.1)		
Married	31(59.6)			23(46.0)		
Bereft of spouse^c^	69(74.2)			56(63.6)		
Employment status		26.77	**0.000**		30.37	**0.000**
Unemployment	40(81.6)			31(64.6)		
Employment	235(58.8)			177(45.3)		
Retired	193(74.2)			167(66.3)		
Losing work abilities	31(81.6)			23(62.2)		
Health insurance^d^		8.03	**0.045**		3.02	0.389
MIUE	24(49.0)			22(44.9)		
NMCS	453(68.2)			361(55.8)		
MIUR	15(68.2)			10(47.6)		
Self-pay^e^	7(58.3)			5(45.5)		
Household size		46.13	**0.000**		25.82	**0.000**
<4	360(75.6)			287(61.7)		
≥8	139(51.3)			111(42.2)		
As % of household income^f^		16.99	**0.000**		4.08	**0.043**
<50 %	230(59.9)			191(50.9)		
≥50 %	269(74.1)			205(58.4)		
Minimum living security household^g^		15.66	**0.000**		1.56	0.212
Yes 93(83.0)				65(60.2)		
No 406(63.9)				333(53.7)		
In-patient service		81.36	**0.000**		50.28	**0.000**
Yes 331(80.9)				265(66.6)		
No 168(49.7)				133(40.3)		

Note: The bold values indicate statistical significance at 5 % level. a: I >10 % of household income, II ≥ 40 % of capacity to pay (non-food expenditure)

b: Of the 747 participants,19 missed household or non-food expenditure data

c: 17 divorced participants were categorized into the group “Bereft of spouse”

d: MIUE: Medical Insurance for Urban Employees Scheme

*NCMS* New Cooperative Medical Scheme

*MIUR* Medical Insurance for Urban Residents Scheme

e: 3 participants covered by commercial insurance were categorized into the group of “self-pay”

f: It means patient annual income as % of annual household income in 2012

g: Minimum living security households were authorized by the local civil affairs department if annual income per person was lower than the minimum living standard

### Determinants of catastrophic health expenditure

Logistic regression yields a wide range of determinants associated with an increased risk of incurring CHE (Table 5). Household factors found to be statistically significant were households with less than four members and those that received minimum living security. Patient factors were unemployment, older age-group, patient incomes accounting for over 50 % of the household income, NCMS membership, and hospitalization.

**Table 5:** Multivariate logistic regression model of determinants of catastrophic expenditure for TB care, China 2012

Variable	Model I^a^			Model II^b^		
	OR _adj_	95 % CI	*P-value*	OR _adj_	95 % CI	*P-value*
Age(years)						
≤40	1.0			1.0		
41–59	2.28	1.31–3.97	**0.004**	1.49	0.88–2.52	0.141
≥60	2.26	1.28–4.54	**0.022**	2.03	1.06–3.88	**0.032**
Residence						
Rural	1.0			1.0		
Urban	0.60	0.24–1.46	0.258	1.49	0.65–3.43	0.342
Education						
None	1.0			1.0		
Primary school	1.03	0.60–1.77	0.913	1.37	0.85–2.21	0.191
Junior school	0.76	0.44–1.33	0.338	0.84	0.52–1.36	0.474
Senior school or above	0.88	0.42–1.82	0.725	0.97	0.51–1.84	0.917
Employment status						
Unemployment	1.0			1.0		
Employment	0.21	0.09–0.49	**0.000**	0.38	0.19–0.76	**0.007**
Retired	0.29	0.11–0.80	**0.016**	0.56	0.24–1.29	0.175
Losing work abilities	0.49	0.14–1.70	0.261	0.58	0.22–1.57	0.285
Health insurance^c^						
MIUE	1.0			1.0		
NMCS	2.55	1.09–5.95	**0.031**	2.57	1.16–5.68	**0.019**
MIUR	2.12	0.62–7.29	0.234	0.86	0.28–2.68	0.800
Self-pay	2.01	0.45–8.86	0.359	1.49	0.35–6.41	0.595
Household size						
<4	1.0			1.0		
≥	0.42	0.28–0.62	**0.000**	0.50	0.34–0.72	**0.000**
As % of household income^d^						
<50 %	1.0			1.0		
≥50 %	2.02	1.35–3.02	**0.001**	1.23	0.86–1.76	0.262
Minimum living security household^e^						
Yes	1.0			1.0		
No	0.47	0.26–0.86	**0.013**	1.12	0.71–1.78	0.629
In-patient service						
No	1.0			1.0		
Yes	5.42	3.71–7.92	**0.000**	3.17	2.27–4.41	**0.000**

Note: The bold values indicate statistical significance at 5 % level. a: I >10 % of household income, II ≥40 % of capacity to pay(non-food expenditure)

b: Of the 747 participants, 19 missed household expenditure or non-food expenditure data, the participants included in Model II is 728

c: MIUE: Medical Insurance for Urban Employees Scheme

NCMS: New Cooperative Medical Scheme

MIUR: Medical Insurance for Urban Residents Scheme

d: It means patient annual income as % of annual household income in 2012;

e: Minimum living security households were authorized by the local civil affairs department if annual income per person was lower than the minimum living standard

## Discussion

TB patients incur high costs for diagnosis and treatment despite the free TB care offered in most settings in China. A recent study analyzed the high costs among multi-drug resistant TB patients in China [25]. This study aimed to estimate the costs associated with and analyze the extent of CHE for TB care in China. It is widely agreed that catastrophic health care expenditure occurs when OOP payments for care force a household to reduce expenditure on basic necessities over an extended period of time [3]. However, there is still no consensus on the formal definition of CHE. Some researchers define CHE as the total health expenditure exceeding a threshold (varying from 5–20 %) of household annual income [3, 5, 28, 29]. Others argue that a measure of the ‘capacity to pay’ (effective income) would better reflect purchasing power than total household income, and define CHE as health payment exceeding a threshold (usually 40 %) of effective income remaining after basic necessities have been met [3, 30]. Many researchers have used household non-food expenditure as a proxy measure for household effective income [9, 30]. In this study, we used two common measures: OOP payments exceeding 10 % of household annual income and OOP payments equaling or exceeding 40 % of household non-food expenditure. Even though both of the definitions are widely used in different studies, there is still no one gold standard for measuring CHE, which highlights the need for validation studies to capture CHE more accurately.

Analysis of the incidence and intensity of TB-related CHE offers an insight into the financial protection that a health care financing system provides for its citizens. It reflects the economic burden shouldered by TB patient households and the financial barriers that may reduce access to TB care. In our study, the incidences of CHE for TB care were 67 % (I) and 55 % (II). Both were higher than the reported rates of 65.0 % (I) and 44.0 % (II) for households of TB patients in Nigeria [30], but slightly lower than the incidence of 78.1 % (I) for households of TB patients in Benin [31]. They were also much higher than those estimated for CHE in general and among non-communicable chronic disease patients in China and other countries [3, 7, 32, 33]. The mean gaps for TB were 40.8 % (I) and 52.1 % (II). Both were much higher compared to 6.0 % (I) and 8.3 % (II) for TB patients in Nigeria, and also much higher than that of 14.8 % (I) for TB patients in Benin. They were also higher than those for general patients in China [34]. Likewise, we also found that MPG for TB patient households measured by the two thresholds were much higher than those estimated in Nigeria. The findings indicate that incidence and intensity of CHE for households of TB patients were both high in China.

Over 45 % of household health care expenditure was spent on hospitalization during TB treatment. This substantial cost was clearly partly due to the high hospitalization rate. In our study, 55 % of patients had been hospitalized during TB treatment, much higher than the ceiling rate of 20 % recommended by some local government TB programs [35, 36]. Reducing this rate may require local health officials to standardize the admission criteria for TB patients and to promote good practice among health providers. Furthermore, a higher reimbursement rate for inpatient health care expenditure might be effective for protecting those TB patients from CHE. Households also pay considerable costs before diagnosis, some 20 % of all OOP payments. This cost was a matter of particular concern because it not only reflects the financial burden on households for obtaining a diagnosis, but may act as a barrier for poor patients to access timely TB care.

Economic status was found to be a key determinant of CHE, consistent with other studies conducted in China and elsewhere [7, 9, 30], with poorer households far more likely to suffer from catastrophic expenditures. Catastrophic health expenditure incidence, using both measures, was highest in the poorest group (Q1). This group also had the highest mean gap and mean positive gap, both much higher than those for the richest group (Q4). Clearly, this finding should be an impetus to the increased provision of pro-poor health insurance and medical assistance policies. Expanding the package for free TB health care among the poor (e.g. including the transport costs occurring in the care-seeking process and ancillary drugs for treatment in the free service package) might be effective for protecting the poor against CHE.

We found that patients covered by NCMS, compared to those in the Urban Employee Basic Medical Scheme (UEBMI), are more likely to experience CHE. One explanation for this phenomenon is that, in general, the reimbursement rate of NCMS is substantially lower than that of UEBMI. Another may be the economic status disparity between rural residents (in NCMS) and urban employees (in UEBMI). We note that the univariate analysis indicates that place of residence (urban/rural) is significantly associated with CHE, but this relationship is not found to be significant in the multivariate analysis. In China, NCMS is designed exclusively for rural residents and UEBMI and Urban Resident Basic Medical Insurance (URBMI) are mainly for urban residents. Place of residence and type of health insurance scheme are thus highly confounded.

As found in other studies, households with four or more members were less likely to experience CHE [7]. One reason might be that there are on average more income earners in such households and thus the impact when one earner falls ill with TB is reduced. This is reflected in our finding that households where the income of the patient accounted for over 50 % of total income had a higher risk of CHE. In relatively small households, which are common in rural China, the illness of a member may not only result in a loss of their income but may reduce the earning potential of other members, who have to provide care and support to the patient and possibly undertake additional household tasks.

This study has some limitations. First, even though we minimized the estimation error by helping the patients in their recall efforts, and also retrieved the health insurance card numbers of the patients when interviewed to check health service records and the expenditures in the health insurance system as far as possible, our measures of annual household income and expenditure, and expenditures on food and health care, relied on self-reported information. This may well have been affected by recall biases. Second, the sample was restricted to TB patients who sought care in local dispensaries and designated hospitals. Many of those who chose not to seek care may have done so due to perceived financial barriers. This probably led to underestimations of the incidence and intensity of CHE.

## Conclusions

This study found that TB is associated with extremely high levels of both the headcount and mean gap measures of CHE. Both were inversely associated with household income level, which indicates that existing health insurance and medical assistance schemes may need to be modified to make them more pro-poor. Though the Chinese government provides a free diagnosis and treatment package for TB patients, the incidence of CHE is much higher than that of illness overall or of NCDs. An integrated policy that expands the free package and ensures UHC, especially the height of UHC, is needed. Hospitalization costs during TB treatment accounted for over 45 % of all OOP payments. There is an urgent need for policies that curb unnecessary hospitalizations and limit inpatient costs for TB patients. This study also identified a number of risk factors for CHE, including age, employment status and household size, which should be taken into account when designing policies to limit the risk of CHE in selected vulnerable groups.

## Additional file

Additional file 1:
**Multilingual abstracts in the six official working languages of the United Nations.** (PDF 369 kb)

## Footnotes

1 Currency exchange rate end 2012: RMB 628 Yuan to US$100.
