# Supplementary material for: Factors that determine catastrophic expenditure for tuberculosis care: a patient survey in China
Source: Infect Dis Poverty. 2016 Jan 25;5:6. doi: 10.1186/s40249-016-0100-6 (PMC4724959; doi:10.1186/s40249-016-0100-6)
Supplement: Additional file 1: — Multilingual abstracts in the six official working languages of the United Nations. (PDF 369 kb) [file 40249_2016_100_MOESM1_ESM.pdf]

Translation of the abstract into the six official working languages of the United Nations

العوامل المُحدّدة للنفقات الكارثية الخاصّة برعاية مرضى السلّ: استطلاع خاص بالمرضى في الصّين

Chengchao Zhou, Qian Long, Jiaying Chen, Li Xiang, Qiang Li, Shenglan Tang, Fei Huang, Qiang Sun, Henry Lucas

ملخص

الخلفيّة: يترتّب عن داء السلّ آثار اقتصادية كارثية تمسّ كلّا من الشخص الذي يعاني من المرض و عائلته. و لقد أجرت العديد من الدراسات تحليلا للنفقات التي يتكبّدها المرضى و أسرهم من أجل تقديم الرعاية الخاصة بهذا المرض. لكن من الواضح أنه لا توجد أيّ دراسة أجرت تقييما لمعدّل و كثافة و العوامل المحدّدة للنفقات الصحيّة الكارثية (CHE) المتعلقة بداء السلّ في الصين. لهذا كانت هذه الدراسة التي بين أيدينا.

الطرق: تمّ أخذ البيانات المستعملة في هذه الدراسة من المسح القاعدي للحكومة الصينية – برنامج Gates Foundation TB Phase II. اشتمل تحليلنا على 747 حالة سلّ. تمّ تقدير النفقات الصحيّة الكارثية الخاصّة بداء السلّ باستعمال طريقتين اثنتين، مع الأسر المُعرّفة على أنها تختبر نفقات صحيّة كارثية (CHE) إذا كانت نفقاتها السنوية على داء السلّ: (أ) تجاوزت 10 بالمائة من مداخيل الأسرة الإجمالية، و (ب) تجاوزت 40 بالمائة من نفقات الأسرة غير المتعلّقة بالغذاء (القدرة على الدفع). تمّ استعمال 'اختبارات تشي مربّع' لتحديد العوامل المرتبطة و تحليل الانحدار اللوجيستي من أجل تعيين العوامل المحدّدة للنفقات الصحيّة الكارثية.

النتائج: كان معدّل النفقات الصحيّة الكارثية **66.8 بالمائة** باستعمال مقياس المداخيل الخاصّة بالأسرة و **54.7 بالمائة** باستعمال النفقات غير المتعلّقة بالغذاء (القدرة على الدفع). تمّت ملاحظة علاقة عكسية بين معدّلات النفقات الصحيّة الكارثية و مستوى مداخيل الأسر. كانت أهمّ العوامل المحدّدة للنفقات الصحيّة الكارثية كالتالي: العمر، حجم المنزل، الوضع الوظيفي، وضع الضمان الصحّي، مدخول المريض كنسبة مئوية من مجمل مداخيل الأسرة، المعالجة في المستشفى و الحدّ الأدنى للأمن المعيشي الأسري. لم تكن للعوامل التي اشتملت على الجنس و الحالة الاجتماعية و نوع حالة السلّ أيّ ارتباطات هامّة بالنفقات الصحيّة الكارثية.

الخلاصات: معدّل النفقات الكارثية الخاصّة برعاية مرضى السلّ في الصّين عالٍ. و هو ما يستوجب سياسة متكاملة لتوسيع حزمة العلاج المجانّي و ضمان تغطية كليّة، و خاصّة زيادة الرعاية الصحيّة المتحدة UHC لمرضى السل. تعتبر أنشطة الحماية المالية و الاجتماعية أمرا ضروريا بالنسبة للمجموعات التي تمّ اعتبارها في حالة خطر.

Translated from English version into Arabic by Mokhtar Oussama Alliouche, through

TRANSLATORS
WITHOUT BORDERS

灾难性卫生支出影响因素研究：一项关于中国结核病人调查分析

周成超，龙倩，陈家应，项莉，李强，汤胜蓝，黄飞，孙强，Henry Lucas

摘要

背景: 结核病通常会给患者及其家庭带来灾难性经济负担。众多研究已对结核病治疗费用情况进行了分析，但很少有研究对结核病引起的灾难性卫生支出发生率、水平及其影响因素进行评价。本研究旨在解决此问题。

方法: 本研究资料来源于中盖结核病二期项目基线调查数据。研究纳入了747例结核病患者。本研究中灾难性卫生支出采用两种测量方法：（1）结核病治疗年花费超过家庭总收入的10%（Ⅰ）；（2）结核病年花费超过家庭非食品支出（支付能力）的40%（Ⅱ）。使用卡方检验对灾难性卫生支出相关因素进行了单因素分析，使用Logistic回归进行了多因素分析。

结果：使用Ⅰ、Ⅱ两种测量方法，灾难性卫生支出发生率分别为66.8%和54.7%。灾难性卫生支出发生率和家庭收入成负相关。灾难性卫生支出相关影响因素包括：年龄、家庭成员数、工作状态、医疗保险、病人收入占家庭收入比重、是否住院及是否低保户等。性别、婚姻状况及结核病类型等因素与灾难性卫生支出无关。

结论：在中国，因结核病引起的灾难性卫生支出发生率水平较高。需要推行一系列政策包括扩大结核病免费服务包、确保结核病患者健康保障覆盖水平尤其是补偿水平，以降低结核病患者及其家庭经济负担。对于结核病患者中发生灾难性卫生支出高危人群需要采取一些针对性经济及社会保护干预措施。

Translated from English version into Chinese by Zhou Chengchou

Facteurs déterminant les dépenses catastrophiques relatives au traitement de la tuberculose : enquête auprès de patients en Chine

Chengchao Zhou, Qian Long, Jiaying Chen, Li Xiang, Qiang Li, Shenglan Tang, Fei Huang, Qiang Sun, Henry Lucas

Résumé

Contexte : La tuberculose a souvent des conséquences économiques catastrophiques tant pour les malades qui en souffrent que pour leur foyer. Plusieurs études ont analysé les dépenses de patients et de ménages en matière de traitement de la tuberculose, mais aucune ne semble avoir évalué l'incidence, l'intensité et les déterminants des dépenses de santé catastrophiques relatives au traitement de la tuberculose en Chine. Tel est l'objectif du présent document.

Méthodes : Les données utilisées pour cette étude sont tirées de l'étude de référence menée par le Gouvernement chinois et la fondation Gates pour le programme TB Phase 2. Notre analyse a pris en compte 747 cas de tuberculose. Nous avons estimé les dépenses de santé catastrophiques relatives à la tuberculose au moyen de deux approches, où il est déterminé que les ménages ont des dépenses de santé catastrophiques si leurs dépenses annuelles moyennes relatives à la tuberculose : (a) dépassent 10 % du revenu total du ménage ; et (b) dépassent 40 % de leurs dépenses non alimentaires (capacité à payer). Des tests X2 ont été utilisés pour identifier les facteurs associés et une analyse de régression logistique a permis d'identifier les déterminants des dépenses de santé catastrophiques.

Résultats : L'incidence des dépenses de santé catastrophiques était de 66,8 % pour la mesure observant les revenus du ménage et de 54,7 % pour la mesure observant les dépenses non alimentaires (capacité à payer). Une association inverse a pu être observée entre les taux de dépenses de santé catastrophiques et le niveau de revenu du ménage. Les principaux déterminants des dépenses de santé catastrophiques sont les suivants : âge, taille du ménage, statut professionnel, statut d'assurance santé, revenus du patient en pourcentage du revenu total du ménage, hospitalisation et statut en tant que ménage ayant une sécurité des conditions de vie minimums. Les facteurs comme le sexe, le statut marital et le type de tuberculose n'ont pas d'association importante avec les dépenses de santé catastrophiques.

Conclusions : L'incidence des dépenses de santé catastrophiques relatives au traitement de la tuberculose est importante en Chine. Une politique intégrée d'expansion du panier de soins gratuits et la mise en place d'une couverture universelle, en particulier le niveau de couverture universelle en santé pour les malades de la tuberculose sont nécessaires. Il est essentiel de mettre en place des interventions de protection sociale et financière pour les groupes identifiés comme présentant un risque.

Translated from English version into French by Claire Michelon, through

Исследование по выявлению факторов, определяющих непомерно большие расходы на противотуберкулезное лечение у пациентов в Китае

Chengchao Zhou, Qian Long, Jiaying Chen, Li Xiang, Qiang Li, Shenglan Tang, Fei Huang, Qiang Sun, Henry Lucas

Аннотация

Базовая проблематика. Противотуберкулезное лечение нередко оказывает катастрофическое влияние на материальное положение лиц, страдающих от данного заболевания, а также их семей. С помощью ряда исследований был проанализирован уровень расходов пациентов и их семей на противотуберкулезное лечение, однако отсутствует какая бы то ни было информация о распространенности, глубине и решающих факторах возникновения непомерно больших расходов на медицинские услуги (CHE), связанные с лечением ТБ, в Китае. Это и станет целью данной работы.

Методы. Данные, используемые в настоящей работе основаны на базовом исследовании правительства Китая и противотуберкулезной программе фазы II Фонда Била и Мелинды Гейтс. Наш анализ охватывает 747 случаев заболевания туберкулезом. Оценка непомерно больших расходов на медицинские услуги, связанные с лечением больных ТБ, осуществлялась с помощью двух подходов при участии семей, испытывающих на себе бремя CHE, с ежегодными расходами на противотуберкулезное лечение в размере: (а) превышающем 10% общего дохода семьи; и (б) превышающем 40% расходов на непродовольственные товары (платежеспособность). Применялись тесты по критерию хи-квадрат для выявления соответствующих факторов и логистический регрессионный анализ для выявления решающих факторов CHE.

Результаты. В первом случае уровень распространения CHE составил 66,8% при использовании показателей доходов семей, во втором — 54,7%, где использовались показатели расходов на непродовольственные товары (платежеспособность). Выявлена обратно пропорциональная зависимость между показателями CHE и уровнем доходов населения. Значимыми факторами возникновения CHE являлись возраст, размер домохозяйства, занятость, статус медицинского страхования, доход больного в процентах от общего дохода семьи, лечение в стационаре и прожиточный минимум семьи. Такие факторы, как пол, семейное положение и форма ТБ не имели никаких существенных связей с CHE.

Выводы. В Китае показатель распространенности непомерно больших расходов на медицинские услуги, сязанные с противотуберкулезным лечением, является высоким. Необходимо внедрение комплексной программы, расширяющей бесплатный пакет лечения и обеспечивающей всеобщий охват, в частности, внедрение единой системы здравоохранения для больных туберкулезом. Существенное значение имеет принятие эффективных мер финансовой и социальной защиты для выявленных групп риска.

Translated from English version into Russian by Anna Romanenko, through

Factores que determinan gastos catastróficos en la atención de la tuberculosis: una encuesta de pacientes en China

Chengchao Zhou, Qian Long, Jiaying Chen, Li Xiang, Qiang Li, Shenglan Tang, Fei Huang, Qiang Sun, Henry Lucas

Resumen

Antecedentes: La tuberculosis (TB) a menudo tiene efectos económicos catastróficos no tan solo sobre el individuo que sufre de la enfermedad sino también sobre en su familia. Varios estudios han analizado los gastos de los pacientes y sus familias en la atención de la TB, pero no parecen haber evaluado la incidencia, intensidad y determinantes del gasto catastrófico en salud con relación a la atención de la TB en China. Ese será el objetivo de este estudio.

Métodos: Los datos utilizados en el presente estudio se obtuvieron de una encuesta de línea de base del gobierno de China - el programa Fase 2 de la Fundación Gates para la TB. Nuestro análisis incluyó 747 casos de TB. Los gastos catastróficos en salud para la atención de la TB se estimaron mediante el uso de dos métodos, considerando que una familia tenía un gasto catastrófico en salud para la atención de la TB si su gasto anual: (A) Excedía el 10% del ingreso familiar total y (b) excedía el 40% de su gasto que no fuese alimento (capacidad de pago). Se llevaron a cabo pruebas del chi cuadrado para identificar factores asociados y un análisis de regresión logística para identificar los determinantes del gasto catastrófico en salud.

Resultados: La incidencia del gasto catastrófico en salud fue del 66,8% si se utilizaba la medición del ingreso familiar y del 54,7% si se utilizaba el gasto que no fuese alimentos (capacidad de pago). Se observó una relación inversa entre los índices de gasto catastrófico en salud y el nivel de ingreso familiar. Los determinantes más importantes del gasto catastrófico en salud fueron: edad, tamaño de la familia, situación laboral, situación en cuanto a seguro médico, ingreso del paciente como porcentaje del ingreso familiar total, hospitalización y condición de familia viviendo en un nivel de seguridad mínimo. Los factores incluidos género, estado civil y tipo de caso de TB no tuvieron relación significativa con el gasto catastrófico en salud.

Conclusiones: Existe una elevada incidencia del gasto catastrófico en salud por atención de TB en China. Se requiere de una política integrada que amplíe los paquetes de tratamiento gratuito y asegure cobertura universal, en particular una mayor cobertura universal de salud para pacientes con TB. Intervenciones en cuanto a la protección financiera y social son indispensables para los grupos de riesgo identificados.

Translated from English version into Spanish by Maria Alejandra Aguada, through
